# What Direct Electrostimulation of the Brain Taught Us About the Human Connectome: A Three-Level Model of Neural Disruption

**DOI:** 10.3389/fnhum.2020.00315

**Published:** 2020-08-07

**Authors:** Hugues Duffau

**Affiliations:** ^1^Department of Neurosurgery, Montpellier University Medical Center, Montpellier, France; ^2^Institute of Functional Genomics, INSERM U-1191, University of Montpellier, Montpellier, France

**Keywords:** awake mapping, direct electrostimulation, human connectome, neural networks, neurophysiology, neuroplasticity

## Abstract

For a long time, the relevance of the information provided by direct electrostimulation (DES) for mapping brain functions was debated. Recently, major advances in intraoperative DES for guiding resection of cerebral tumors in awake patients enabled the validation of this method and its increased utilization in basic neurosciences. Indeed, in addition to the cortical stimulation used for many decades in epilepsy surgery, axonal mapping was developed thanks to DES of the white matter tracts, giving original insights into the neural connectivity. Moreover, functional results collected during intrasurgical mapping have been correlated with neuropsychological performances before and after DES-guided resection, and with perioperative neuroimaging data. Thus, it was evidenced that DES offers the unique opportunity to identify both cortical and subcortical structures *critical* for cerebral functions. Here, the first aim is to propose a three-level model of DES-generated functional disruption, able to explain the behavioral consequences elicited during awake surgery, i.e., (i) DES of an input/output unimodal (e.g., somatosensory or motor) network inducing “positive” responses (as involuntary movement); (ii) DES of a distributed specialized network inducing a within-system disruption leading to specific “negative” disorders (e.g., exclusive language deficit with no other disorders); (iii) DES generating an inter-system disruption leading to more complex behavioral disturbances (e.g., the inability to perform dual-task while each function can be performed separately). Second, in light of this model, original findings gained from DES concerning the human connectome, complementary to those provided by functional neuroimaging (FNI), are reviewed. Further longitudinal multimodal investigations are needed to explore neuroplasticity mechanisms.

## Introduction

Since its popularization by Penfield (1954), intraoperative direct electrostimulation (DES) has regularly been used during brain resection to detect eloquent areas, especially for epilepsy surgery (Ojemann et al., 1989). Beyond the clinical benefit which consists in minimizing the risk of permanent neurological deficits, pioneering works proposed new models of cerebral anatomo-functional organization based upon the DES findings, e.g., the sensorimotor homunculus (Penfield and Boldrey, 1937) or a mosaic distribution of language sites (Ojemann et al., 1989). However, the relevance of the information provided by DES for mapping brain functions was debated for many decades, because: (i) these data were collected in brain-damaged patients, who often experienced preoperative functional disturbances—notably in intractable epilepsy; (ii) DES is an invasive method; and (iii) mechanisms underlying DES were poorly understood, particularly with a bias related to possible cortical spreading. Therefore, because of the emergence of non-invasive functional neuroimaging (FNI) techniques, the place of DES was marginalized in fundamental neurosciences.

Recently, major advances in intraoperative DES for guiding resection of cerebral tumors in awake patients changed the way neuroscientists think of stimulation mapping. This technique is increasingly used in patients who undergo early surgery, thereby with only mild or even no preoperative functional disturbances—as in the incidental discovery of slow-growing low-grade glioma (Duffau, 2018). Thus, a normal cognitive assessment before surgery allows a more extensive neuropsychological mapping throughout the resection. Furthermore, in addition to the cortical stimulation utilized for many decades in epilepsy surgery (commonly with extra operative mapping using grids), axonal mapping was developed using DES of the white matter tracts (Duffau, 2015). By gathering findings gained from both cortical and subcortical stimulation, accuracy, reliability, and reproducibility of DES mapping were dramatically improved. Indeed, functional results collected during intrasurgical mapping have been correlated with neuropsychological performances before and after DES-guided resection. Such correlations enabled to validate DES method, by demonstrating that the introduction of new cognitive tasks during awake mapping resulted in a minimization of the persistent postsurgical deficits in the domain monitored intraoperatively, e.g., decrease of complex movement disorders by mapping the bimanual coordination during surgery (Rech et al., 2014), or decrease of mentalizing disturbances by mapping theory of mind during resection (Herbet et al., 2015a). Importantly, DES offers the unique opportunity to identify cortical and subcortical structures *critical* for cerebral functions, especially because axonal DES provides original insights into the direct functioning of neural connectivity (Duffau, 2015). These correlations between DES and cognitive scores have also permitted a better understanding of the neural foundations underpinning sensorimotor, visuospatial, language, cognitive, and emotional processing, leading to new models of dynamic anatomo-functional architecture (Herbet and Duffau, 2020).

Here, the first aim is to propose a three-level model of DES-generated functional disruption, able to explain the behavioral consequences elicited during awake surgery. Second, in light of this model, original findings gained from DES concerning the human connectome and its plastic potential are reviewed: these data evidence that DES mapping currently plays a pivotal role in basic neurosciences.

## A Three-Level Model of DES Neural Disruption

DES can modify the activity of a population of neurons by changing the voltage gradient across the neuronal membrane: when the current crosses cells, it can modulate their membrane potential and trigger neuronal responses (Vincent et al., 2016). Therefore, DES is helpful to identify the functional role of each brain area stimulated, which should nonetheless be conceived only as a part of a more distributed neural network. Indeed, every structure responsive to DES is an input gate into a large-scale circuit rather than an isolated discrete eloquent structure (Mandonnet et al., 2010). Therefore, by generating a “virtual transitory dysfunction,” DES detects the cortical hubs and white matter fascicles which are critical for brain function by disrupting a subnetwork for a few seconds (Duffau, 2015). Interestingly, the same functional responses are reproduced when repeated DES is applied over the same site. In surgical practice, such behavioral interferences guide the resection, by pursuing the tumor removal until functional boundaries have been reached in awake patients, allowing an increase of the extent of resection while minimizing postoperative neurological morbidity, even in areas traditionally considered as essential in a localizationist dogma (Duffau, 2014).

Besides its clinical implication, DES can also shed light on the networking organization of the brain. However, although any kind of inappropriate response induced by DES represents sufficient information for surgical purposes (since the resection is stopped as soon as neurological deficits are elicited to avoid sequelae), it is more difficult to draw robust neuroscientific conclusions about the neural architecture without an optimized understanding of mechanisms underpinning the large spectrum of transient disturbances caused by DES. To clarify this wide variety of transitory dysfunctions, a three-level model of DES-generated functional disruption is proposed, based upon two types of troubles:

– a “positive response,” which is defined as the induction by DES of an unexpected neurological response in a patient at rest (as involuntary movement, tingling or phosphenes)
– a “negative trouble,” which is defined as a functional disturbance (whatever its nature, that is, from complete arrest to response error) evoked by DES while the patient is performing a task, this interference within the neural network preventing him/her transitorily to achieve successfully the on-going task.

The first “basic” level corresponds to the DES of an input network, which is the first relay of information entering the brain, or to DES of an output network, which is the last relay sending information outside the brain (Ius et al., 2011). The input circuits include the thalamocortical tracts running to the primary somatosensory cortex and the optic radiations running to the primary visual cortex, whereas the output circuit is composed of the primary motor cortex and the cortico-spinal tracts. When DES is applied at their level, it generates a “positive” response, namely, paresthesias during stimulation of the primary somatosensory system (more rarely feeling of heaviness of the limb or fading limb), phosphenes during stimulation of the primary visual system, and involuntary movement during stimulation of the primary motor system. In surgical practice, the patient should be at rest when DES is performed. Such basic responses seem to be because these networks, composed of a cortical area and its projection fibers, are mainly unimodal (Ius et al., 2011).

The second “intermediate” level consists of DES of distributed function-specific networks, composed of multiple delocalized cortical areas interconnected by associative white matter pathways. These functional systems subserve movement control, spatial cognition, language, working memory, or emotion (Duffau, 2015). DES of a specialized network induces a within-system disruption leading to specific “negative” troubles, namely, with task inhibition. Because these networks are constituted by several parallel and interactive subnetworks, DES may cause a discrete deficit related to the disruption of one subcircuit, or a more global deficit due to the disruption of the entire system. For example, concerning the language system, stimulating specifically the dorsal stream mediated by the arcuate fasciculus during a naming task elicits phonological paraphasia; stimulating specifically the ventral stream mediated by the inferior fronto-occipital fasciculus (IFOF) evokes semantic paraphasias; whereas stimulating both pathways [especially at their junction underneath the posterior temporal areas or dorsolateral prefrontal cortex (dlPFC)] causes complete anomia due to disruption of the whole network (Duffau et al., 2014). Importantly, no non-language deficit is generated simultaneously, e.g., the patient is still able to move despite transient DES-elicited language disorders. In surgical practice, the patient should be asked to perform the appropriate task according to the sub-network that the neurosurgeon wants to map depending on the tumor location. For instance, if a naming task is achieved to monitor language semantic processing into the contact of the left IFOF in a left-hander with right hemispheric lateralization of language, no semantic paraphasia will likely be induced with a high risk to damage the ventral stream because of “false negative.” Nonetheless, if the patient is asked to achieve a non-verbal semantic association task, semantic disturbances will probably occur during the DES of the same neural structure allowing the preservation of the pathway devoted to multimodal semantic processes (Moritz-Gasser et al., 2013). Combining intraoperative neurophysiologically-sophisticated methods, namely, DES and electrocorticographic recording of cortico-cortical evoked potentials (Kunieda et al., 2015) and subcortical-cortical evoked potentials (Yamao et al., 2014) could be of utmost interest to better investigate the mechanisms underpinning DES neural disruption (Vincent et al., 2020).

**Figure 1:**
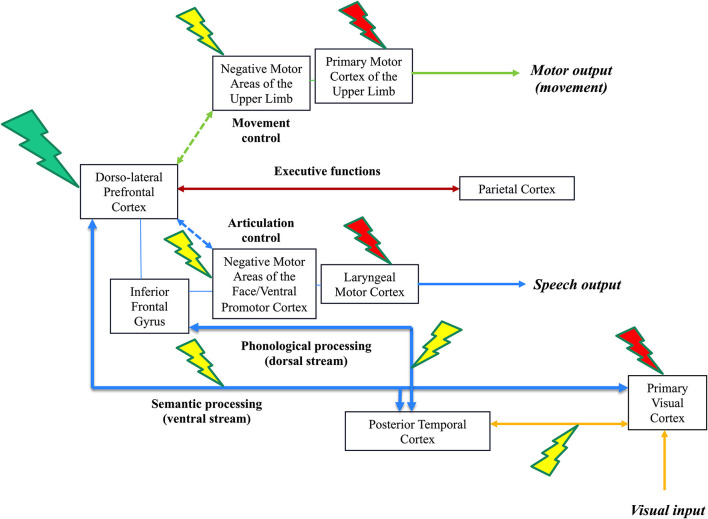
Illustration of the three-level model of direct electrostimulation (DES) neural disruption: red lightning bolt symbolizes the first level (input/output disruption), yellow lightning bolt symbolizes the second level (within-network disruption) and green lightning bolt symbolizes the third level (inter-network disruption). This is a schematic representation of the visual system (lower, connectivity in yellow), speech and language system (lower, connectivity in blue), executive functions system (middle, connectivity in brown) and motor system (upper, connectivity in green) in the left hemisphere of an awake patient who performs a dual-task associating picture naming and movement of the right upper limb. DES of the primary visual system induces phosphenes (1st level, a red lightning bolt at the level of the optic tracts and/or primary visual cortex), while DES of the occipito-temporal connections induces visual hemiagnosia [2nd level, a yellow lightning bolt over the posterior inferior longitudinal fasciculus (ILF)]. In parallel, the patient can continue to move the right upper limb. In the speech and language system, DES of the laryngeal motor cortex elicits involuntary vocalization (1rst level, red lightning); DES of the ventral premotor cortex and frontoparietal articulatory loop (not shown) elicits speech apraxia/arrest (anarthria); while DES of the dorsal phonological route induces phonemic paraphasia and DES of the ventral semantic route induces semantic paraphasia (2nd level, red lightning). Of note, stimulating both dorsal and semantic streams may generate complete anomia. In parallel, the patient can continue to move the right upper limb. In the motor system, DES of the primary motor cortex of the upper limb and its corticospinal tracts (motor execution, output) causes an involuntary movement (1st level, red lightning); DES of the negative motor cortex (movement control) and its fibers (not shown) generates an arrest of movement (2nd level, yellow lightning). In parallel, the patient can continue to perform the picture naming task. During DES of the dorsolateral prefrontal cortex (integrative hub), the brain is not able to coordinate its networks (including the frontoparietal connections involved in the executive functions and then the goal maintenance), the patient cannot achieve dual-task anymore: the patient is only capable to perform each task separately, naming or upper limb movement, but not both simultaneously (3rd level, green lightning).

The third “integrated” level consists of an inter-system disruption evoked by DES, that is, disturbances in the interactions between function-specific networks (e.g., between language and working memory), leading to more complex behavioral disturbances, such as the inability to perform several tasks simultaneously (multi-tasking). Neural processing cannot be conceived in a segregated account, with parallel circuits acting in isolation: complex cognitions at the service of adaptive, context-specific behaviors emerge from spatiotemporal dynamic interactions between the specialized functional systems. Such integration should transitorily be generated to succeed in cognitive demanding, functionally multi-determined behavior tasks (Herbet and Duffau, 2020). An example of complex cognitive activities is dual-tasking, in which the brain must coordinate its networks to reach the task goal. In surgical practice, the patient is regularly asked to perform a movement of the upper limb simultaneously with a picture-naming task. With no DES, a cross-system interaction between the language (semantics, phonology, and articulation), motor (movement control and execution), and executive functions (goal maintenance) networks are needed to achieve the task efficiently thanks to integrative hubs such as the dlPFC. As mentioned above (second level of DES disruption), when the language system is impaired by DES, the patient can maintain the task goal, but only the motor task is performed; while when the motor system is disturbed, the patient is still able to maintain the task goal, but only the naming task is achieved. Interestingly, the third level of DES disruption can be illustrated by the impairment of dlPFC as an entry window to a complex inter-system integration: the brain has difficulties to coordinate its networks and the patient cannot achieve dual-task anymore, he/she is only able to perform each task separately but not conjointly (Figure 1).

This three-level model of DES functional disruption offers several clinical and neuro-scientific advantages. From a surgical perspective, it allows the optimal selection of tasks during surgery and how to administrate them (order, sequence, combination) throughout the resection, based upon the better understanding of DES mechanisms. Indeed, beyond the symptom itself, these mechanisms of neural disruption can be deciphered into the operating theater, enabling a more accurate interpretation of the awake mapping in real-time to make the best decision to optimize the tumor removal while preserving a high level of quality of life—as defined by the patient him(her)self before surgery, according to his/her needs, job, hobbies and lifestyle (Herbet and Duffau, 2019). In other words, this model can be helpful to plan awake surgery in order not only to avoid severe permanent deteriorations as hemiplegia or aphasia but also to spare networks subserving higher-order cognitive and emotional functions, as well as their dynamic interactions, to preserve behavior and to enable a return to an active familial, social and professional life. From a fundamental perspective, this model permits a refined analysis of DES-derived responses to investigate the human connectome uniquely. Indeed, findings collected during transient network interferences induced by DES provide unprecedented direct access into the actual neural dynamics.

## Reunifying Clinical DES Brain Mapping and Basic Neurosciences

Arguments supporting that DES mapping in tumor surgery allows valid conclusions on the localization of human brain functions have already been detailed (Duffau, 2011). Furthermore, DES offers two major interests in comparison with the commonly used FNI to study the human connectome. First, although functional MRI is an indirect reflection of the areas involved in a given brain function, without the possibility to distinguish those participating vs. those essential to this function, stimulation mapping allows the identification of the structures critical for neural functions—i.e., generating permanent behavioral consequences if damaged (Sarubbo et al., 2020). Second, whereas tractography enables a reconstruction of the anatomical trajectory of white matter tracts using biostatistics modeling, axonal DES is capable to give direct information regarding the function of the subcortical connectivity (Duffau, 2015). Consequently, in light of the three-level model of DES-neural disruption, the bridge between surgical DES mapping and fundamental neurosciences can be reinforced, by translating the findings issued from brain-damaged patients to improved knowledge of the physiology of the human central nervous system, and by proposing new models of dynamic connectivity (Herbet and Duffau, 2020).

In a first step, structural-functional correlations gained from intraoperative DES and neuropsychological assessments provided original insights into the neurobiology of function-specific network. For example, regarding movement and visual function, DES of the output primary motor cortex and pyramidal tracts evokes involuntary motor responses, and DES of the optic tracts or primary visual cortex provokes basic positive responses (e.g., phosphenes) or more complex visual illusion (e.g., metamorphopsia), respectively (1st level; Ius et al., 2011). At a higher level, DES of the so-called “negative motor areas” and its subcortical pathways (such as the frontostriatal tract) generates arrest of ongoing movement, possibly with interruption of complex bimanual coordination due to a disruption of the network devoted to movement control (2nd level; Rech et al., 2019). Similarly, DES of the posterior inferior longitudinal fasciculus (ILF) that connects the visual cortex and fusiform gyrus may generate visual hemiagnosia during a picture-naming task because of disturbances of the object recognition abilities (2nd level; Fernández Coello et al., 2013). At the most integrated level, DES of plurimodal hubs, such as dlPFC implicated in executive functions, may prevent to perform movement and visual picture naming task simultaneously (3rd level, inter-system disruption; Herbet and Duffau, 2020).

Concerning speech and language, DES of the laryngeal motor cortex and its projection fibers induces uncontrolled vocalization (1st level; Dichter et al., 2018); within-system DES of the ventral premotor cortex and articulatory loop generates anarthria or DES of the basal temporal areas and anterior ILF causes lexical access deficit (2nd level; Herbet et al., 2016b); while DES of the prefrontal cortex and superior longitudinal fasciculus (SLF) may elicit control disturbances in switching from one language to another one in multilingual patients—due to disruption between executive system and each subnetwork sustaining each language (3rd level; Moritz-Gasser and Duffau, 2009). Here, the goal is not to exhaustively review the neural foundations of every functional system that have previously been pooled in original probabilistic atlases of essential cortico-subcortical structures, based upon DES-driven responses in awake patients (Tate et al., 2014; Sarubbo et al., 2020). Rather, the main purpose is to highlight the multilevel organization of segregated and interactive networks revealed by DES. Indeed, taken as a whole, these original data have recently led to the proposal of a meta-networking theory of brain functions, relying on transitory changes of relationship within and across neural circuits, resulting in a perpetual succession of new equilibrium states reflecting moment-to-moment environmental demands—as well as to more long-lasting modifications of network properties, including use-dependent neuroplasticity (Herbet and Duffau, 2020).

A step forward, in this meta-networking view of brain processing, potentiating neuroplastic dynamics can sustain not only the human propensity to learn complex abilities and to be creative but also the brain capacity to compensate for neural loss after cerebral insult. An atlas of neuroplasticity has been elaborated based on neurological recovery (or not) following brain tumors surgery (Herbet et al., 2016a). Interestingly, a parallel can be made between this atlas, which identified structures with low, intermediate, and high potential of functional compensation, and the 3-level model of intraoperative DES neural disruption. Indeed, input and output neural networks that generate “positive” responses when stimulated correspond to the main foundations of the “minimal common brain,” namely, the structures where compensatory mechanisms are the most limited (Ius et al., 2011). In the event of injury, the risk of a severe and irrevocable deficit, such as hemiplegia or hemianopia, is very high. Interestingly, the interindividual variability of these structures is very low (Duffau, 2017). Concerning the structures where DES causes a specific disturbance due to a within-specialized network disruption (second level), the potential of recovery is higher, especially for cortical areas. Although a lesion of associative pathways (e.g., surgical injury of long-distance white matter bundles) has a high risk to generate a permanent disconnection syndrome, such as conduction aphasia if the left arcuate fasciculus is damaged, hemineglect if the right SLF is damaged, or semantic disorders if the IFOF is damaged (Herbet et al., 2015c), a focal cortical lesion might be (at least partly) compensated by the rest of the distributed network. Recovery is nonetheless possible if the highly integrative cortical hubs, such as the dlPDF, and their subcortical connectivity have been preserved, resulting in more subtle deficits, regardless the domain—e.g., difficulties in complex bimanual movement (Rech et al., 2014); increase of reaction time for lexical access; slight impairment of working memory (du Boisgueheneuc et al., 2006); or mild impairment of mentalistic processes such as subjective empathy (Herbet et al., 2015b). These mechanisms of cerebral reallocation are possible in a connectomal account of neural distribution, explaining recovery following resection of tumors within regions deemed inoperable in a rigid localizationist framework, as Broca’s area or Wernicke’s area (Duffau, 2014). Of note, the interindividual anatomo-functional variability of these structures is high (Duffau, 2017). Finally, regarding the third level, disruption of the integrative process between conation, cognition and emotion may lead to more complex behavioral changes, impacting, for example, the personality (at the extreme with a risk to generate various neurological or psychiatric diseases, possibly due to the decompensation of borderline traits) or creativity (Herbet and Duffau, 2020). Therefore, beyond the simplistic standard neurological examination, even an extensive neurocognitive evaluation performed according to the current guidelines could not be sensitive enough to objectively reveal such an unbalance in the metanetwork. More sophisticated behavioral tasks should be developed to assess more subtly brain-damaged patients with neuropsychological performances wrongly considered as “normal.”

## Limitations

This study may have some limitations.

First, DES is an invasive technique, then available in the limited clinical situation. However, to prevent any deleterious effects, DES consist of stimulating the surface of the exposed brain, conversely to electro-microstimulation (EMS) used in animals, which activates neurons by applying a current through microelectrodes implanted in the parenchyma. This is the reason why EMS became the preferred method for modulating neural activity and demonstrating functional properties in animal experiments (Histed et al., 2013). Therefore, in humans, a combination of DES with non-invasive stimulation mapping method as transcranial magnetic stimulation (TMS) may be of great interest. Nonetheless, in a recent study which compared navigated repetitive TMS with intraoperative DES in brain tumor patients, TMS had only 81.6% sensitivity, 59.6% specificity, 78.5% positive predictive value and 64.1% negative predictive value for preoperative language mapping (Motomura et al., 2020), confirming that DES remains the gold standard.

Second, it is unclear how the extent of the whole-brain network is inhibited by local block for axon using DES. Nevertheless, if the repetitive stimulation is <200 Hz, γ-aminobutyric acid-related inhibition seems to prevent the propagation of electrostimulation beyond the first synapse (Logothetis et al., 2010): thus, DES may inform us about its effect on a network’s functional status when only a part of this network is stimulated.

Third, data gained from DES come from the pathological model, with a possibility that reorganization has already occurred in the brain network of tumor patients, especially with low-grade glioma. However, gathering many patients’ data into the database can compensate these potential confounding factors: therefore, DES findings in glioma patients have been shown to represent a valid model for exploring normal brain functions (Herbet and Duffau, 2020). Indeed, in a recent functional atlas of critical neural circuits based upon 1,821 DES responses in 256 glioma patients, the overall distribution of the functional responses was topographically congruent with the current literature using FNI (Sarubbo et al., 2020), including large meta-analysis involving healthy human subject (Zhang et al., 2010; Thiebaut de Schotten et al., 2011). Of note, to make full use of the original findings provided by DES, very detailed knowledge of cortical and subcortical anatomy is needed for the precise stimulation mapping (Vincent et al., 2016).

## Conclusions and Perspectives

DES is a reliable tool to map brain functions, complementary to FNI, since providing unique data regarding the indispensability of cerebral structures, especially concerning the neural connectivity. These original findings can be translated from brain-damaged patients to the modeling of the physiological functioning of nervous system processes. The next step is to combine intraoperative neurophysiology, i.e., DES and electrocorticographic recording of cortico-cortical and axonal-cortical evoked potentials (to refine the understanding of DES neural disruption; Vincent et al., 2020), with pre- and post-operative optimized cognitive and behavioral assessments as well as with longitudinal structural and FNI, to decipher the pathophysiological mechanisms underlying neuroplasticity. Preliminary serial multimodal studies evidenced morphometric (Almairac et al., 2018) and functional reorganization of the human connectome, notably with contralesional homotopic compensation (Vassal et al., 2017), and with changes of the connectivity between the cortex, the deep gray nuclei and the cerebellum (Boyer et al., 2016). Beyond brain mapping, perspectives could be to actively modulate such dynamic intra-system and inter-system integration using non-invasive neural electrostimulation, particularly by using TMS.

## Author Contributions

HD conceived the idea and wrote the manuscript.

## Conflict of Interest

The author declares that the research was conducted in the absence of any commercial or financial relationships that could be construed as a potential conflict of interest.
